# Microenvironment mediated alterations to metabolic pathways confer increased chemo-resistance in CD133^+^ tumor initiating cells

**DOI:** 10.18632/oncotarget.10838

**Published:** 2016-07-26

**Authors:** Alice Nomura, Patricia Dauer, Vineet Gupta, Olivia McGinn, Nivedita Arora, Kaustav Majumdar, Charles Uhlrich III, Joseph Dalluge, Vikas Dudeja, Ashok Saluja, Sulagna Banerjee

**Affiliations:** ^1^ Division of Basic and Translational Research, Department of Surgery, University of Minnesota, Minneapolis, MN, USA; ^2^ Division of Surgical Oncology, Department of Surgery, Sylvester Comprehensive Cancer Center, University of Miami, Miami, FL, USA; ^3^ Department of Chemistry, Mass Spectrometry Laboratory, University of Minnesota, Minneapolis, MN, USA

**Keywords:** CD133, tumor initiating cells, metabolism, hypoxia, ROS

## Abstract

Chemoresistance in pancreatic cancer has been attributed to tumor-initiating cells (TICs), a minor sub-population of tumor cells. However, the mechanism of chemo-resistance in these cells is still unclear.

In the current study, immunohistochemical analysis of *LSL-Kras*^G12D^; *LSL-Trp53*^R172H;^ *PdxCre* (KPC) murine tumors indicated that hypoxic regions developed through tumor progression. This hypoxic “niche” correlated with increased CD133^+^ population that had an increased HIF1A activity. Consistent with this observation, CD133^+^ cells had increased glucose uptake and activity of glycolytic pathway enzymes compared to CD133^−^ cells. Mass spectrometric analysis (UPLC-TQD) following metabolic labeling of CD133^+^ cells with [^13^C]-U6 glucose confirmed this observation. Furthermore, although both populations had functionally active mitochondria, CD133^+^ cells had low mitochondrial complex I and complex IV activity and lesser accumulation of ROS in response to standard chemotherapeutic compounds like paclitaxel, 5FU and gemcitabine. CD133^+^ cells also showed increased resistance to all three chemotherapeutic compounds and treatment with Glut1 inhibitor (STF31) reversed this resistance, promoting apoptotic death in these cells similar to CD133^−^ cells.

Our study indicates that the altered metabolic profile of CD133^+^ pancreatic TIC protects them against apoptosis, by reducing accumulation of ROS induced by standard chemotherapeutic agents, thereby confering chemoresistance. Since resistance to existing chemotherapy contributes to the poor prognosis in pancreatic cancer, our study paves the way for identifying novel therapeutic targets for managing chemoresistance and tumor recurrence in pancreatic cancer.

## INTRODUCTION

Pancreatic cancer is a devastating disease with more than 44,000 cases per year in United States alone. Tumor recurrence, aggressive biology and resistance to available therapy contribute to its grim survival rates [1]. Tumor initiating cells (TIC) are a rare population of cells within a tumor, which are largely attributed to both tumor relapse as well as drug resistance in this disease. Though chemo-resistance is considered a hallmark of TICs, the mechanisms of chemo-resistance in these cells are not fully understood. The TICs in pancreatic cancer have been isolated and studied using a number of different surface markers like CD133 [2, 3], CD44, CD24, ESA [4], c-met [5] ALDH [6]. Along with the surface markers, pancreatic cancer TICs have been known to have increased chemo-resistance [7, 8], increased expression of survival genes [3] and anti-apoptotic genes. Though a number of studies have been focused on isolation and characterization of TICs, not a lot of research has been directed towards understanding the metabolic pathways in a TIC. Recent studies suggest that TICs may have special metabolic properties that distinguish them from the bulk of tumor cells, and that such biochemical properties may constitute a basis for developing new therapeutic strategies to eliminate these cells [9].

Cancer cells are characterized by rapid uptake of glucose. Unlike normal cells, that require growth factor signaling to utilize available resources for every cellular processes, cancer cells display a deregulated metabolism, that relies on anabolism [10]. Thus most of the energy in these cells is spent on biosynthetic reactions required for rapid differentiation, proliferation and growth. Metabolic intermediates of glycolysis and glutaminolysis get shunted to biosynthetic pathways required for cellular growth. Carbon and nitrogen from glucose and glutamine are utilized for nucleoside and amino-acid synthesis, whereas pyruvate enters the citric acid cycle to facilitate fatty acid synthesis by supplying acetyl- and malonyl-CoA. This dysregulated cellular metabolism is often responsible for resistance mechanisms of cancer cells to chemotherapeutic compounds [11–15].

Similar to normal stem cells, the TICs also possess self-renewal and differentiation properties. [16]. It is well known that undifferentiated embryonic stem cells as well as adult stem cells have different energetic metabolism as compared to fully differentiated cells. While stem cells rely mostly on anaerobic metabolism, oxidative phosphorylation (OXPHOS) is the preferred energy metabolism pathway in fully differentiated cells [17]. Thus, the bioenergetic pathways between cancer cells and human embryonic stem (hES) cells are thought to be analogous. Prior to implantation and vascularization *in vivo*, hES cells are in a hypoxic environment between 1% to 5% of oxygen [18]. Under these conditions, they remain pluripotent. As hypoxic embryonic cells cannot produce adequate amounts of ATP via mitochondrial OXPHOS, they rely on anaerobic metabolism to produce ATP to meet their energy requirements [19], just as many cancer cells do [20–23]. Further, hypoxic conditions in these cells results in activation of HIF1 mediated signaling, leading to increased glucose uptake and glycolysis [24].

To date, energy metabolism in TICs has been sparsely explored. In fact, it is unknown whether the metabolic profile of CSCs is similar to that observed in embryonic stem cells. Recent studies using cultured cancer stem cells *in vitro* have reported that these “stem cells” have increased oxidative phosphorylation [25]. However, it is also accepted that the tumor microenvironment in the course of tumor progression is responsible for creation of the appropriate niche, resulting in enrichment of stem-like tumor initiating population [26]. The metabolic phenotype of CSCs appears to vary across tumor types. While in breast cancer and nasopharyngeal carcinoma CSCs were found to be predominantly glycolytic [27–29], CSCs in glioma and glioblastoma [30, 31], lung cancer [32], and leukemia [33] appear to rely on mitochondrial OXPHOS.

In addition to the lack of energy metabolism mechanisms in tumor initiating cells, how these altered metabolic pathways in a TIC actually contribute to its chemo-resistance has also not been studied. Previous studies from our group have shown that CD133^+^ cells are a reliable representation of pancreatic TICs and these cells recapitulate almost all the properties of a TIC. A follow-up study also revealed that an overexpression of CD133 in a pancreatic cancer cell line leads to increased tumorigenesis and invasion [34]. Further, CD133^+^ population also had increased expression and activity of ABC transporter genes resulting in chemo-resistance to standard chemotherapeutic agents like Gemcitabine, Paclitaxel and 5FU [3]. CD133^+^ cells also showed increased expression of anti-apoptotic genes like Bcl-2 and Survivin [3].

Based on these observations, we have now studied the metabolic pathways in the CD133^+^ pancreatic TICs and compared them with CD133^−^ non-TICs. In the current study we show that CD133^+^ TIC in pancreatic cancer are enriched in hypoxic regions of the tumor and have increased HIF1 activity. They also have an increased glucose uptake and increased glycolysis. We further show that these cells have low mitochondrial activity in spite of having physiologically healthy mitochondria. Our results also show that this altered metabolism in pancreatic TIC also confers a survival advantage to these cells by lowering ROS accumulation, thereby leading to a chemo-resistance phenotype.

## RESULTS

### CD133^+^ cells are present in hypoxic niches in the pancreatic tumor

**Figure 1:**
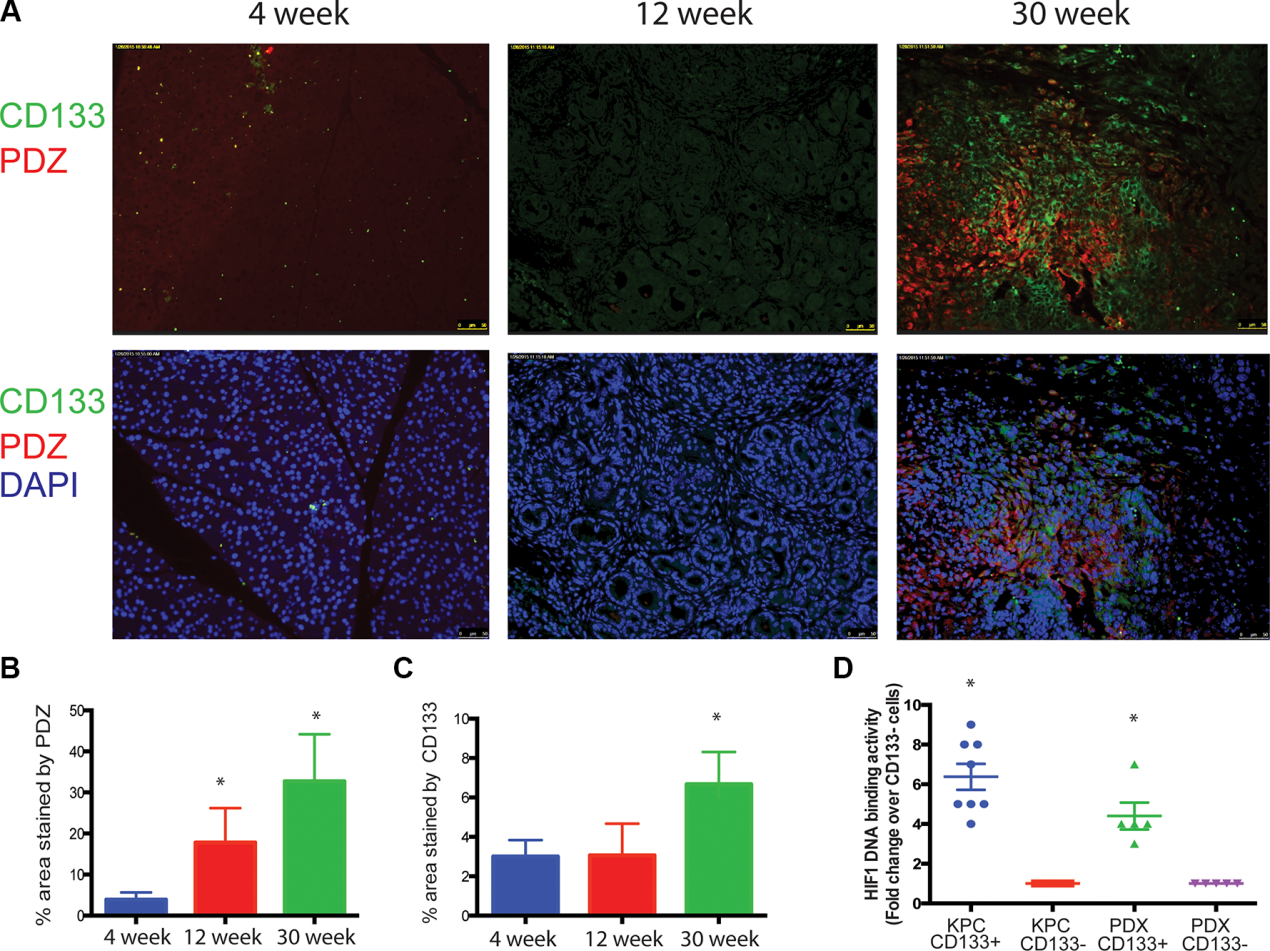
Hypoxia enriches for CD133^+^ cells in pancreatic cancer Hypoxic regions stained with Pimonidazole showed a correlation with CD133 expression in KPC tumors during tumor progression (**A**). Percentage of area stained with PDZ (**B**) and CD133 (**C**) was calculated using Image J software. CD133^+^ cells from KPC tumors and patient tumor derived xenografts (PDX) had increased HIF1 activity (**D**). The * represents *p* < 0.05.

Pancreatic tumors are known to be extremely hypoxic. To study if CD133 expression in KPC tumors correlated with the hypoxic areas, we injected KPC mice with pimonidazole (marker for hypoxia) and co-stained slides with CD133. Pimonidazole (PDZ) staining co-localized with the CD133 staining in these tumors (Pearsons Coeff. 0.69) indicating that hypoxic areas indeed had increased population of pancreatic TIC (Figure 1A–1C; Supplementary Figure S1). To confirm if CD133^+^ TICs indeed had increased HIF1A DNA binding activity, we performed an ELISA based DNA binding assay for HIF1A protein in the nuclear extracts of CD133^+^ and CD133^−^ cells from the KPC tumors (Figure 1D, *n* = 6–7). HIF1A binding was significantly increased in CD133^+^ cells confirming that CD133^+^ cells co-localized to the hypoxic areas in the tumor and had increased HIF1A activity.

### CD133^+^ cells have increased glucose uptake leading to increased glycolysis

**Figure 2:**
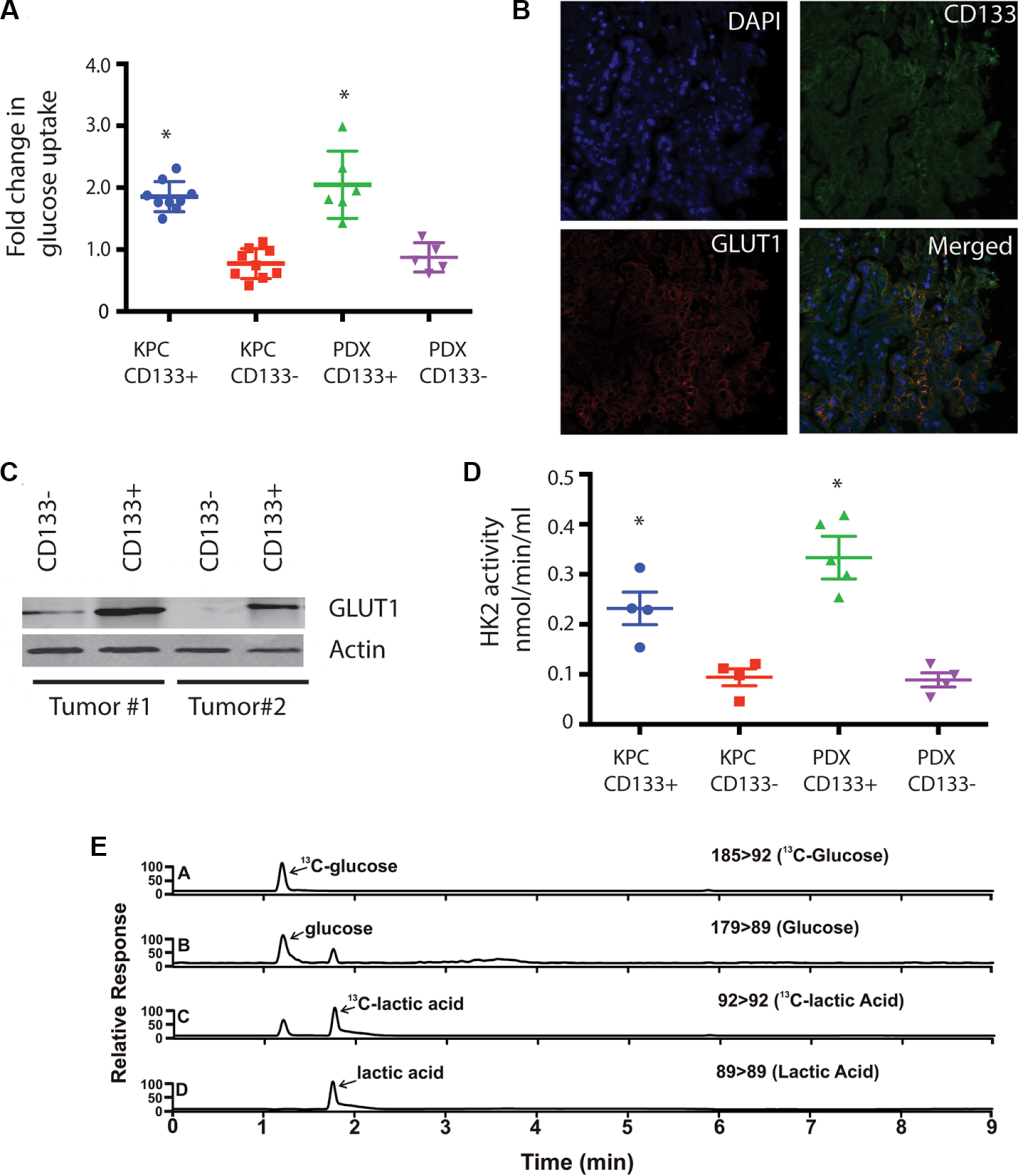
CD133^+^ cells have increased glycolytic activity. CD133^+^ cells had increased uptake of glucose (**A**) compared to CD133^−^ cells. They had increased expression of GluT1 as seen by immunofluorescence of CD133/GLUT1 (**B**) and western blot (**C**). This was consistent with increased hexokinase activity (**D**). Metabolic labeling CD133^+^ cells with 13C glucose followed by UPLC-TQD confirmed this observation (**E**). The * represents *p* < 0.05.

Hypoxia drives an increased glucose uptake in cancer cells resulting in increased glycolysis. To address this, we next analyzed CD133^+^ tumor initiating cells from KPC mouse tumors as well as human patient derived xenografts (PDX) in SCID mice for the glucose uptake using 2-NBDG, a fluorescently-labeled deoxyglucose analog, as a probe for the detection of glucose taken up by cells. CD133^+^ cells had increased glucose uptake compared to the CD133^−^ population in both tumor types (Figure 2A). This was further corroborated when CD133^+^ cells showed an increased expression of GLUT1 compared to CD133^−^ population from both KPC and PDX tumors (Figure 2B, 2C). To see if this increased glucose uptake influenced the glycolytic activity of CD133^+^ cells, we studied the expression of glucose metabolizing genes in CD133^+^ and CD133^−^ cells.

In concurrence with increased expression and activity of glucose transporters, CD133^+^ cells also had increased expression of glycolytic genes (Supplementary Figure S2A). To confirm if this increased gene expression also correlated with increased glycolytic activity, we assayed for HK2 activity in CD133^+^ and CD133^−^ population. Consistent with the increased expression of glycolytic genes, CD133^+^ cells had 3-fold increased activity for HK2 enzyme (Figure 2D). Additionally, labeling CD133^+^ cells with ^13^C-^6^Glucose and following the label using UPLC-TQD, confirmed increased glucose uptake by CD133^+^ cells (Figure 2E).

### CD133^+^ cells had increased LDH activity leading to lactate production

**Figure 3:**
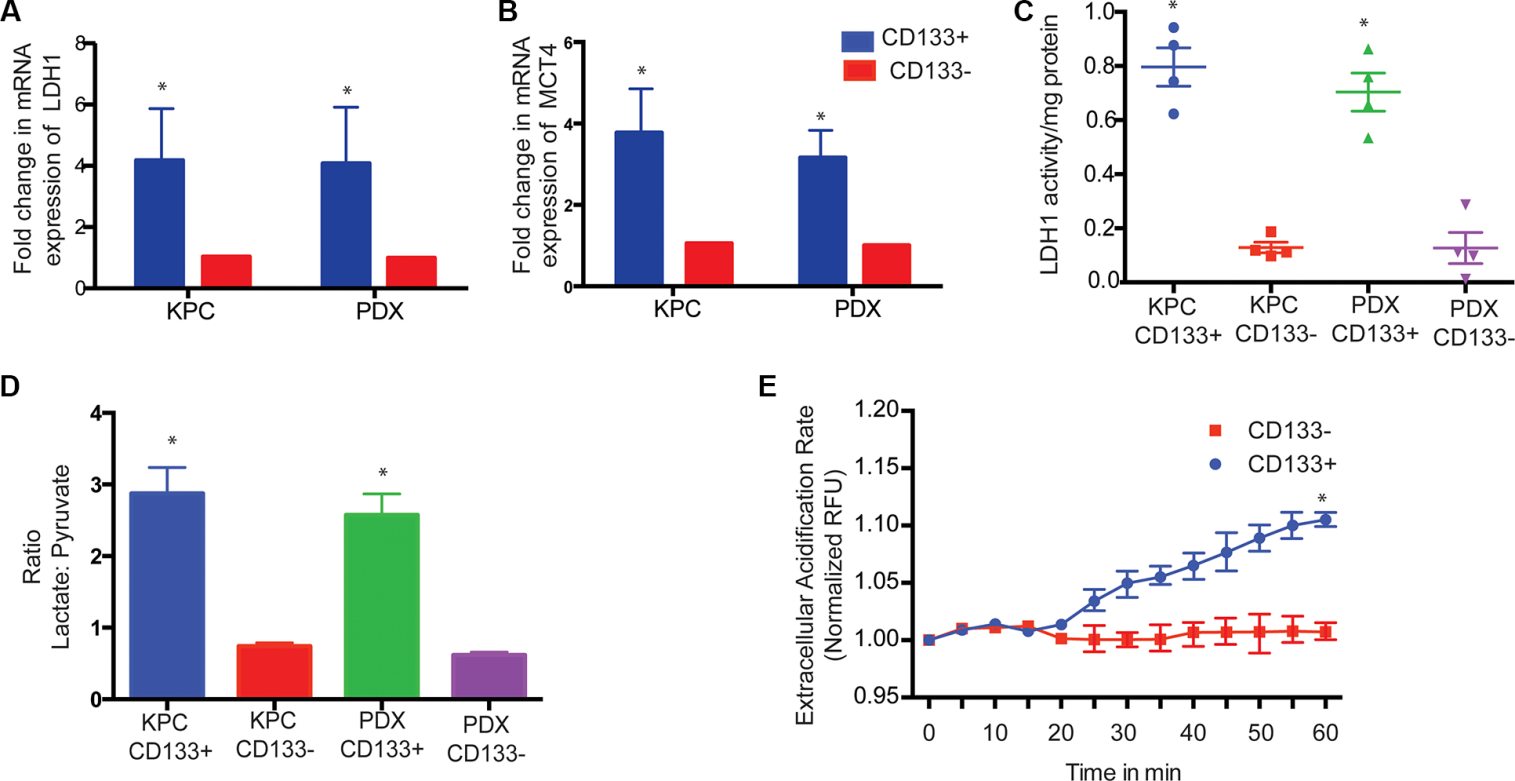
CD133^+^ cells have increased LDH expression and activity LDH1 (**A**) and MCT4 (**B**) expression was increased in CD133^+^ cells. Activity of LDH was also higher in CD133^+^ cells (**C**) resulting in a skewed lactate:pyruvate ratio (**D**). CD133^+^ cells also showed increased MCT4 activity as measured by extracellular acidification rate, ECAR (**E**). The * represents *p* < 0.05.

The high rates of aerobic glycolysis in the tumor cells result in increased LDH activity leading to production of high levels of lactate/H^+^ (lactic acid), which must be exported from the cell. Thus, to maintain the enhanced glycolytic flux and intracellular physiological pH, tumor cells upregulate pH regulators, such as monocarboxylate transporters (MCTs), which perform the efflux of lactic acid into the extracellular microenvironment. This prevents intracellular acidosis and subsequent cell death [35, 36]. To see if LDH and MCT gene expressions were altered in CD133^+^ and CD133^−^ cells isolated from multiple KPC tumors and PDX tumors, we studied the expression of these two genes. Both LDH1 and MCT4 mRNA were increased in CD133^+^ cells compared to CD133^−^ cells (Figure 3A, 3B). Consistent with the above observation, tissue sections of PDX also showed significant co-staining of LDHA/CD133 (52% events) and MCT4/CD133 (56% events) (Supplementary Figure S3A). LDH enzyme activity was also increased in CD133^+^ cells (Figure 3C). This was also consistent with our observation that CD133^+^ cells had increased HIF1A activity, as both LDHA and MCT1-4 are downstream targets of HIF1A.

Since LDH catalyzes the conversion of pyruvate to lactate and back, we determined the lactate/pyruvate ratio in the CD133^+^ cells. CD133^+^ cells had increased lactate production compared to pyruvate production having a lactate:pyruvate ratio of 2.8 compared to CD133^−^ cells that had a lactate:pyruvate ratio of 0.72 (Figure 3D). This was further confirmed in our UPLC-TQD studies after labeling cells with ^13^C-6 Glucose (Figure 2E, Supplementary Figure S3B).

Transport of lactate outside the cells via mono-carboxylate transporters can be measured by ECAR or extracellular acidification rate. CD133^+^ had an increased ECAR compared to CD133^−^ cells in KPC tumors (Figure 3E). ECAR in CD133^+^ cells was indeed mediated via monocarboxylate transporters as inhibiting these transporters using different doses of CHC significantly decreased ECAR in CD133^+^ cells from multiple KPC primary tumors (Supplementary Figure S3D).

We next silenced CD133 in S2-VP10 cells that have a 3–4% CD133^+^ cells to determine whether the increased glycolysis was due to CD133 expression. As expected, silencing CD133 resulted in decreased HK2 activity and LDH activity (Supplementary Figure S4A, S4B).

### TCA cycle is independent of glycolysis in CD133^+^ cells

**Figure 4:**
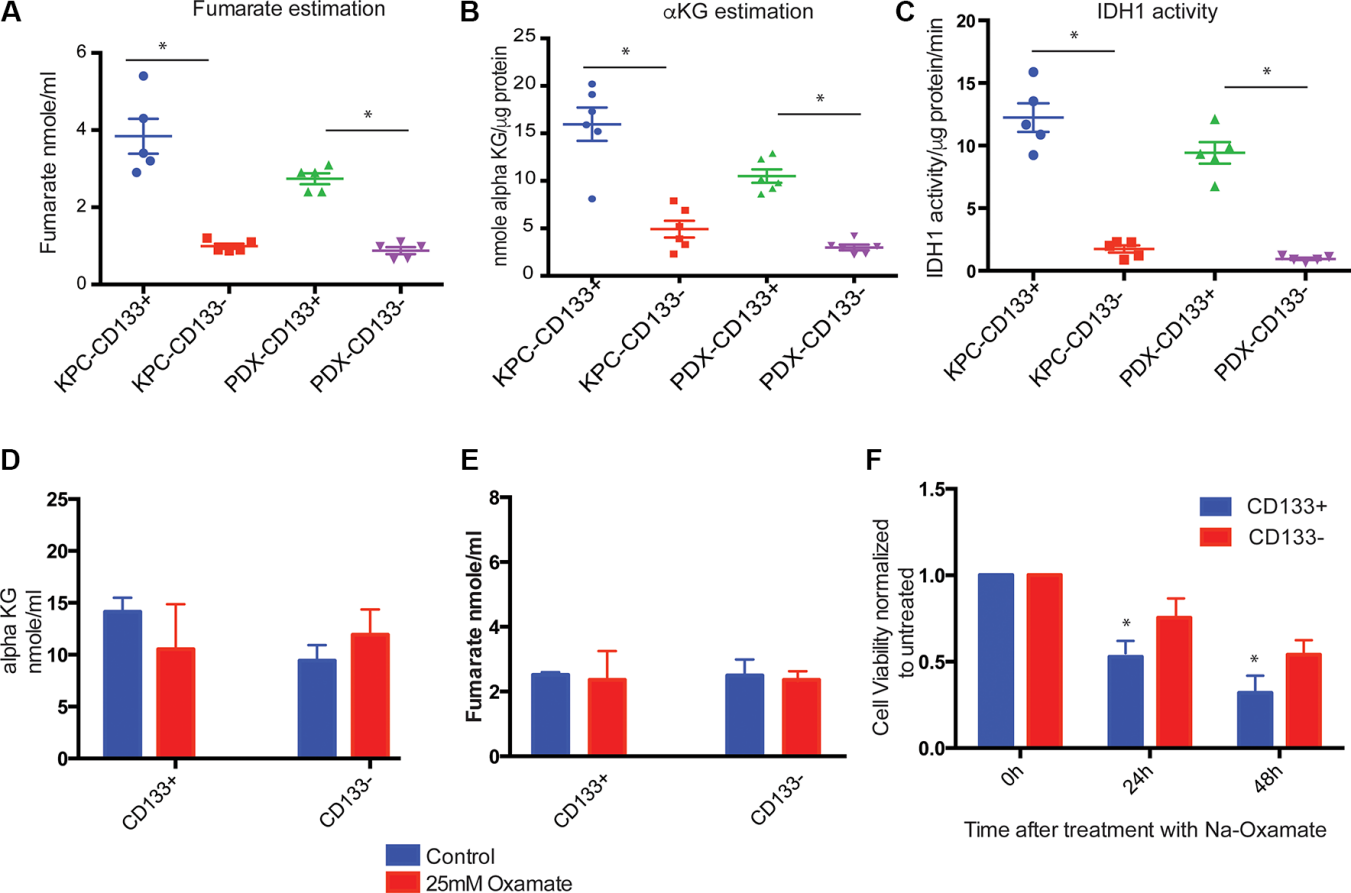
TCA cycle in CD133^+^ cells CD133^+^ cells had increased TCA cycle intermediates like fumarate (**A**) and alpha ketoglutarate (**B**). IDH1 activity was also increased in these cells (**C**). Upon inhibition of glycolysis with Na-Oxamate, there was no significant change in the TCA cycle intermediates fumarate (**D**) or alpha ketoglutarate (**E**). Inhibition of glycolysis with sodium oxamate resulted in decreased viability of CD133^+^ cells (**F**). The * represents *p* < 0.05.

Under aerobic conditions, pyruvate produced by the glycolysis reaction is converted to acetyl CoA, which enters the TCA cycle and generates the FADH2 and NADH required for oxidative phosphorylation. Since CD133^+^ cells produced less pyruvate compared to lactate, we studied the TCA cycle enzymes and their functions in these cells to see if this pathway was impaired. Our study showed that TCA cycle enzymes were overexpressed in CD133^+^ cells compared to CD133^−^ cells (Supplementary Figure S2B). When TCA cycle intermediates were measured, CD133^+^ cells showed increased production of fumarate and alpha keto-glutarate compared to CD133^−^ cells (Figure 4A, 4B). To evaluate if these cells had increased IDH1 activity, we assayed the activity of this enzyme (Figure 4C). CD133^+^ cells had 8–10 fold increased IDH activity. This was consistent with our previous observation that CD133^+^ cells has increased expression of IDH1 gene [3].

To determine how TCA cycle in CD133^+^ cells was being fueled, we first inhibited LDH using Na-oxamate. Na-Oxamate is a substrate analog of pyruvate and inhibits LDH by competing with pyruvate preventing its entry into the mitochondria. As expected, treatment with Na-oxamate resulted in an accumulation of pyruvate in both CD133^+^ as well as CD133^−^ cells (Supplementary Figure S3C). However, treatment with Na-oxamate for 3 h did not hinder the progression of TCA cycle in CD133^+^ or CD133^−^ cells and no significant difference was observed in the alpha keto-glutarate or fumarate synthesis (Figure 4D, 4E). A longer treatment with Na-oxamate (24 h and 48 h) resulted in decreasing viability of both CD133^+^ as well as CD133^−^ cells (Figure 4F). This indicated that the TCA cycle in the PDAC cells was not dependent on pyruvate for acetyl CoA synthesis. However, inhibition of glutaminase using Compound 968 resulted in decreased accumulation of alpha-ketoglutarate (Supplementary Figure S5) in CD133^+^ cells.

### CD133^+^ cells maintain mitochondrial morphology but show less mitochondrial electron transport chain (ETC activity)

**Figure 5:**
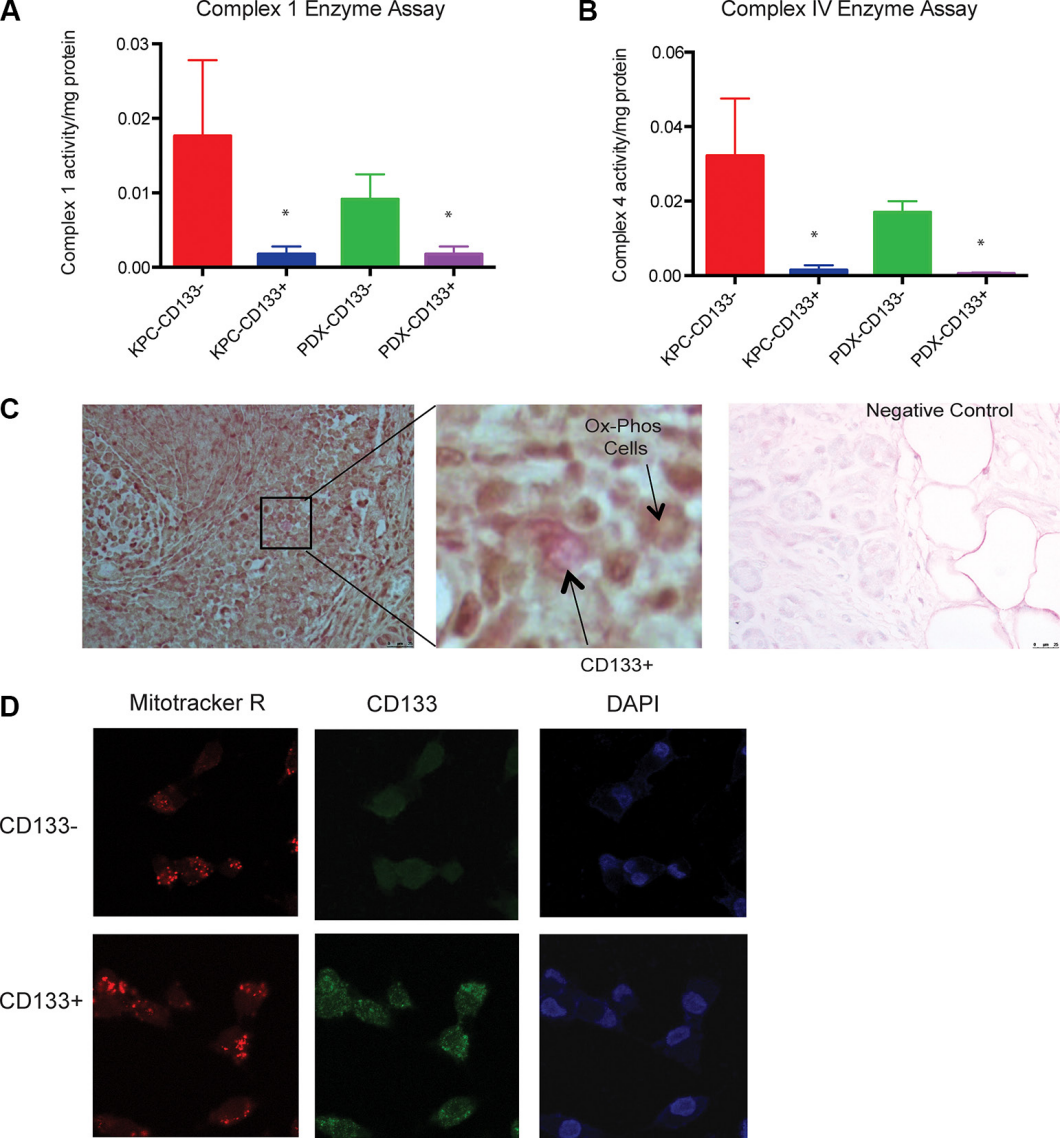
CD133^+^ cells had less mitochondrial activity Mitochondrial complex 1 (**A**) or complex 4 (**B**) activity was very low in CD133^**+**^ cells compared to CD133^−^ cells. In an *in situ* complex IV assay, cryosections were incubated with cytochrome C and DAB in the presence of catalase. The active cytochrome C oxidase (COX) in the CD133^−^ cells actively oxidized the cytochrome C resulting in dark coloring of the tissue, while CD133^−^ cells (magnified field) did not show the brown coloration. Cryosections treated with 1% Na-Azide was used as a negative control (**C**). Both CD133^+^ and CD133^−^ population had similar numbers of functional mitochondria as visualized by uptake of mitotracker Red (**D**). The * represents *p* < 0.05.

To study if the increased glycolytic activity resulted in reduced mitochondrial activity in the CD133^+^ TIC, we assayed for mitochondrial enzyme complexes in these cells. CD133^+^ cells had much decreased Complex I activity as well as Complex IV activity (Figure 5A, 5B). We also observed that CD133^+^ cells had less oxygen consumption rate (OCR) compared to CD133^−^ cells (Supplementary Figure S6). Since the tumor microenvironment is the primary determinant of metabolism in these cells, we performed an *in situ* cytochrome oxidase assay (Complex IV) on flash frozen tumor tissues and co-stained these sections for expression of CD133. This assay clearly showed that CD133^+^ cells did not co-stain with the cells rapidly undergoing oxidative phosphorylation (stained brown). Cryosections treated with 1% Na-Azide were used as a negative control (Figure 5C). To rule out the probability of mitochondrial damage while isolation of CD133^+^ cells from tumors, we studied the mitochondrial morphology of these cells by staining cells with Mitotracker Red. Mitotracker red is a viable mitochondrial dye that is taken up by the mitochondria of healthy cells. CD133^+^ and CD133^−^ cells showed identical Mitotracker Red staining confirming the integrity of mitochondria both cell types (Figure 5D).

### Altered energy needs in CD133^+^ population induce resistance to cell death in response to cytotoxic drugs

Decreased mitochondrial activity in quiescent cells is considered to be an evolutionarily conserved survival benefit as it confers protection to cells from adverse effects of cytotoxic drugs. Since the CD133^+^ cells showed decreased mitochondrial activity, we hypothesized that this metabolic shift in the tumor initiating cells was required for inhibition of apoptosis and resistance to known cytotoxic drugs. Previously published data from our lab show that CD133^+^ cells have increased resistance to chemotherapeutic agents like gemcitabine, 5FU and paclitaxel [3].

**Figure 6:**
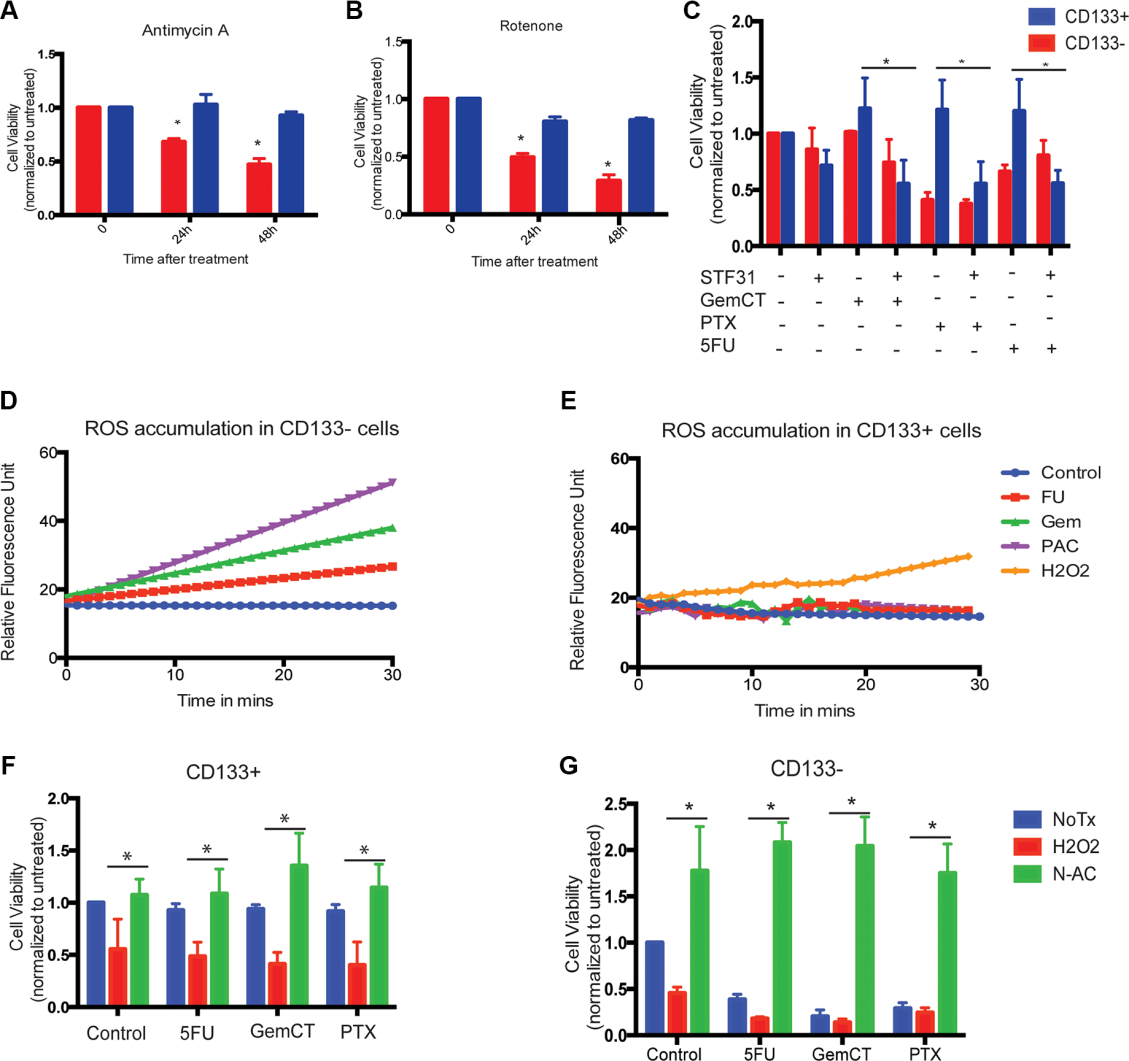
Low mitochondrial activity resulted in survival advantage of CD133^+^ cells CD133^**+**^ cells were resistant to mitochondrial poison Antimycin A (**A**) or Rotenone (**B**). Blocking glycolysis with 2.5 uM STF31 resulted in sensitizing CD133^+^ cells to Gemcitabine (500 nM), 5FU (1 uM) and Paclitaxel (1 uM) (**C**). CD133^−^ cells accumulated ROS when treated with cytotoxic drugs Gemcitabine, 5FU and Paclitaxel (**D**), while same treatment did not result in accumulation of ROS in CD133^+^ cells (**E**). Addition of exogenous “ROS inducer” H2O2 resulted in decreased cell viability in CD133^+^ cells indicating that low ROS accumulation was responsible for chemo-resistance in these cells, addition of N-acetyl cysteine, a ROS inducer, rescued cells from H2O2 induced cell death (**F**). In same experiment, CD133^−^ cells showed increased sensitivity to chemotoxic drugs and cell viability was rescued when ROS was blocked with N-acetyl cysteine. The * represents *p* < 0.05.

To confirm that low mitochondrial activity was indeed responsible for reduced cell death, we treated both CD133^+^ and CD133^−^ cells with mitochondrial poisons like Rotenone and Antimycin A. Interestingly, treatment of CD133^+^ cells with both Antimycin A and Rotenone also resulted in decreased cell death in CD133^+^ population compared to the CD133^−^ population (Figure 6A, 6B).

In contrast, when glycolysis in these cells was inhibited using either STF31, a GLUT1 inhibitor, the CD133^+^ cells showed an increased sensitivity to cytotoxic agents like Gemcitabine, paclitaxel and 5FU (Figure 6C), resulting in extensive cell death. This indicated that the altered metabolic pathways indeed catered to a survival advantage in CD133^+^ tumor initiating cells in pancreatic cancer resulting in a chemo-resistance phenotype.

To see if the above resistance was owing to decreased accumulation of ROS in these cells in response to the cytotoxic agents, we used H2DCF to test for ROS accumulation in response to treatment with Gemcitabine, 5FU and paclitaxel. While CD133^−^ cells showed an extensive accumulation of ROS following treatment, CD133^+^ cells showed little or no accumulation of ROS following treatment. This indicated that low mitochondrial activity was preventing the accumulation of ROS in these cells (Figure 6D, 6E).

Further, exogenous induction of ROS by treating cells with H2O2 reverted the resistance to cell death in CD133^+^ cells, while effect on cell viability of CD133^−^ cells remained unchanged in both H2O2 treated and untreated cells (Figure 6F, 6G).

Similarly, when both CD133^+^ and CD133^−^ cells were treated with ROS inhibitor N-acetylcysteine, prior to treatment with cytotoxic agents, CD133^+^ cells showed no significant change in cell viability when compared to those treated with Gemcitabine, paclitaxel and 5FU where as CD133^−^ cells showed significantly increased rescue from cell death after treatment with these chemotherapeutic drugs (Figure 6F, 6G).

### Metabolic alteration results in increased efflux of chemotherapeutic agents

**Figure 7:**
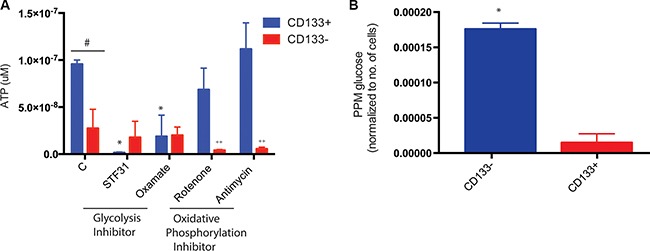
ATP in CD133^+^ cells CD133^**+**^ cells made more ATP than CD133^−^ cells. Inhibition of glycolysis resulted in less ATP production in CD133^+^ cells while inhibition of oxidative phosphorylation had no effect on ATP production. On the contrary, CD133^−^ cells inhibition of oxidative phosphorylation resulted in decreased ATP production (**A**). Stoichiometric analysis showed CD133^+^ cells had more glucose uptake than CD133^−^ cells, leading to more ATP production (**B**). The * represents *p* < 0.05 in treated CD133^+^ cells compared to untreated, # represents *p* < 0.05 in CD133^+^ vs CD133^−^ cells, ++ represents *p* < 0.05 in treated CD133^−^ cells compared to untreated.

Our previous studies shows that CD133^+^ pancreatic TICs have an increased expression and activity of ABC transporters. These transporters are fueled by ATP, and actively pump our chemotherapeutic compounds from a tumor cell thereby maintaining a sub-lethal concentration of drugs in the cell. The active mitochondria are the powerhouse of the cells and are primarily responsible for ATP generation. When tested for ATP production, CD133^+^ cells isolated from KPC cell lines as well as KPC tumors showed almost one log higher ATP production compared to CD133^−^ cells (Figure 7A). This was perplexing since the CD133^+^ cells did not have mitochondrial activity. To see if the ATP in the CD133^+^ cells was being generated at the glycolysis level by substrate level phosphorylation, we inhibited glycolysis using GLUT1 inhibitor STF31 and LDH inhibitor sodium oxamate. Interestingly, inhibition of GLUT1 decreased ATP accumulation in CD133^+^ cells while inhibition of LDH did not have any effect on ATP accumulation (Figure 7A). This indicated that ATP was indeed being produced by substrate level phosphorylation during glycolysis. To confirm this, we performed a stoichiometric analysis of glucose uptake in CD133^+^ cells vs CD133^−^ cells using UPLC-TQD. Our results showed that CD133^+^ cells had almost 8–9 fold more glucose uptake compared to CD133^−^ cells (Figure 7B).

## DISCUSSION

Increasing evidence demonstrates that CSCs are protected and regulated by a specialized tumor microenvironment niche, which plays a crucial role in the maintenance of the CSC biological properties, including self-renewal, differentiation, invasion, metastasis, therapeutic resistance, and genetic instability [37]. Although the CSC niche is still poorly understood, one of the most important components in this niche is hypoxia [11, 38]. In pancreatic cancer, as the tumor develops, the pressure from the fibro-inflammatory stroma constrict the blood vessels within it, leading to low oxygen concentration and resulting in a hypoxic niche [39–41]. These micro-environmental signals are believed to be responsible for enrichment of CSC population in a tumor.

Similar to the ways in which hypoxia maintains the physiological functions of normal stem cells [7], recent advances have shown that hypoxic stress also plays a critical role in the maintenance of CSCs in solid tumors [8–10]. Research into breast cancer shows that hypoxic tumors induced by anti-angiogenic agents contain a significantly higher percentage of CSCs [11], and prostate cancer data indicate that prostate cancer cells under hypoxic conditions possess greater stem-like properties [12]. Similarly, ovarian cancer cells under hypoxia upgrade their stem-like properties through the upregulation of stemness-related factors and behave more aggressively when returned to a higher oxygen environment [13].

Our findings show that as the tumor progresses, hypoxic regions develop within the tumor and this indeed correlates with the CSC marker CD133 expression in these tissues (Figure 1A–1C). Further, CD133^+^ cells from tumors derived from KPC mice as well as PDX show increased HIF1A activity compared to the CD133^−^ cells (Figure 1D).

Since hypoxia and stabilized HIF1A results in increased glucose uptake and glycolysis, we studied the glucose uptake in CD133^+^ vs CD133^−^ cells. Our results indicated that CD133^+^ cells had an increased glucose uptake, leading to an increased glycolysis compared to CD133^−^ cells. Key glycolytic enzymes like HK2 not only showed increased expression in these cells, but also showed increased activity. High glycolysis in CD133^+^ cells, however, did not lead to an increased pyruvate production. CD133^+^ cells also had increased LDH activity, resulted in an increased production of lactate. Labeling CD133^+^ cells with ^13^C-C6 glucose, in an attempt to track it through glycolysis by a UPLC-TQD confirmed the observation that CD133^+^ cells not only had increased uptake of glucose but most glucose taken up by these cells was also being converted to lactate, resulted in a skewed lactate: pyruvate ratio. Moreover, increased lactate transport via mono-carboxylate transporters (MCT1-4) in the CD133^+^ cells resulted in an increased extracellular acidification in these cells. Inhibition of mono-carboxylate transporter with CHC, an inhibitor, decreased extracellular acidification rate. Interestingly, extracellular acidification by lactate has been associated with increased stemness in breast cancer [42].

In aerobic glycosylation, the pyruvate produced as a result of glycolysis enters the TCA cycle in the mitochondria. However, TCA cycle can also be fueled independent of pyruvate. In tumor cells, glutaminolysis feeds the TCA cycle. We observed that in CD133^+^ cells the TCA cycle appeared to be uncoupled from the glycolysis. Inhibition of glucose uptake (by apigenin or STF31) did not alter the activity of enzymes involved in the TCA cycle in CD133^+^ cells. Similarly, inhibition of lactate dehydrogenase in CD133^+^ cells by sodium oxamate did not change TCA cycle enzymes, even though all these treatments (STF31, apigenin and sodium oxamate)decreased viability and induced apoptosis in CD133^+^ cells. However, when glutaminase was inhibited, the accumulation of TCA cycle intermediates decreased in CD133^+^ cells (Supplementary Figure S5). This indicated that in CD133^+^ pancreatic TICs the TCA cycle was being fueled by glycolysis independent mechanism, namely glutaminolysis.

We next focused on the mitochondrial electron transport chain in the CD133^+^ and CD133^−^ cells from the pancreatic tumors. Mitochondrial electron transport is mediated by 4 enzyme complexes. The mitochondrial Complex I (NADH:ubiquinone oxidoreductase) catalyzes the reaction in which electrons are removed from NADH and transferred to a lipid-soluble carrier, *ubiquinone* (Q). The reduced product, ubiquinol (QH_2_), freely diffuses within the membrane, and Complex I translocates four protons (H^+^) across the membrane, thus producing a proton gradient. Our studies showed that CD133^+^ cells have very low complex I activity compared to CD133^−^ cells. We next checked the mitochondrial complex IV activity (a cytochrome C oxidase) in order to confirm if CD133^+^ cells indeed lacked mitochondrial activity. As seen in complex 1, CD133^+^ cells had almost no complex IV activity. Mitochondrial morphology, however, was preserved in both CD133^+^ and CD133^−^ cells. Though recently published reports indicate that CD133^+^ pancreatic TICs have increased oxidative phosphorylation, these TICs have been cultured *in vitro* under “spheroid” conditions [25]. It is unlikely that under these conditions the appropriate hypoxic niche is maintained in these cells. In our experiments performed on the tumor cell lysates or on cryosections of tumors where the tumors have been minimally exposed to the laboratory environment, we observe a distinct upregulation of GLUT1 (Figure 2B), LDHA and MCT4 expression (Supplementary Figure S3), indicating an increased glucose uptake and lactate production and efflux. In addition, our *in situ* complex IV assay confirms that CD133^+^ cells do not have active oxidative phosphorylation pathways (Figure 5C).

Since complex I is one of the main sites at which superoxide production occurs and hence is responsible for production of reactive oxygen species (ROS), we studied ROS accumulation in CD133^+^ cells and compared them with CD133^−^ cells. Our results showed that ROS production was indeed low in CD133^+^ cells compared to CD133^−^ cells. When CD133^+^ and CD133^−^ cells were treated with cytotoxic concentrations of Gemcitabine, 5FU and Paclitaxel (classically associated with ROS production), CD133^+^ cells did not show any ROS accumulation, whereas CD133^−^ cells showed an increased production of ROS (Figure 6). These treatments further resulted in CD133^−^ cell death where as CD133^+^ cells were not affected. This was consistent with our previously published observation that CD133^+^ cells were resistant to standard chemotherapeutic agents. This cell death was indeed mediated by ROS, as inhibition of accumulated ROS with N-acetyl cysteine rescued the CD133^−^ cells from chemotherapeutic agent mediated death (Figure 6). The low mitochondrial activity in CD133^+^ cells thus conferred a survival advantage by protecting cells from ROS accumulation.

Inhibition of glycolysis by either blocking Glut1 by STF31 or by inhibition of LDH activity by sodium oxamate resulted in decreased resistance of CD133^+^ cells to chemotherapeutic agents like gemcitabine, 5FU and paclitaxel. This observation indicated that the altered metabolic state in CD133^+^ cells was indeed contributing to its chemo-resistant phenotype. Increased glycolysis leading to an increased production of lactate resulted in increased lactate transport into the tumor microenvironment, causing extracellular acidification. This probably skewed the pH balance in the cell preventing passive diffusion of the drug into the cells. Our previous results indicate that CD133^+^ cells have increased ABC transporter activity. These transporters act as drug efflux pumps and actively transport drugs out of the cells, thereby preventing a build-up of lethal concentrations of the drug inside the cell. Interestingly, it is also reported that these transporters are regulated by HIF1A [43]. It is possible that the increased HIF1A in the CD133^+^ cells is driving the expression of these transporters.

Though cancer metabolism and its role in the biology of the disease are gaining importance, there is no in-depth study on the metabolic pathways in tumor initiating cells. Our study for the first time has demonstrated how the metabolic pathways in pancreatic tumor initiating cells may not only differ from the metabolic pathways in the bulk tumor cells, but also how these altered bioenergetics confer chemo-resistance to this population.

Pancreatic tumors are notoriously chemo-resistant. Further, the recurrence of these tumors results in a dismal survival statistics. Tumor initiating cells are reported to be responsible for both recurrence and chemo-resistance in this disease. Thus successful therapeutic intervention in this disease is only possible once we understand the underlying mechanisms of chemo-resistance. Our study shows that understanding the metabolic pathways in TIC and non-TIC populations in pancreatic cancer may hold the key to developing therapies that can completely abrogate the TIC population by overriding their chemo-resistant mechanisms.

## MATERIALS AND METHODS

### Plasmids and cell lines

Human cDNA CD133 expression plasmid (EX-Z0396-M02) and empty vector plasmid (EX-NEG-M02) were obtained from GeneCopoeia. Lentiviral shRNA pGIPZ vectors; NS (RHS4348) and aCD133 (V2LHS_71816) were obtained from Thermo Scientific.

MIA PaCa-2 (ATCC) and stable MIA-derivatives were maintained in DMEM (Hyclone) containing 10% fetal bovine serum. S2-VP10 cells were cultured in RPMI 1640 (Hyclone) supplemented with 10% fetal bovine serum. Stable clones were selected and maintained in Geneticin (Invitrogen) and Puromycin (Clontech) for MIA PaCa-2 and S2-VP10 derivatives, respectively.

### Isolation and labeling of CD133^+^ tumor initiating cells from tumors

The CD133^+^ population was separated from the mouse progenitor cells and other CD133^−^ cells using MACS separation (Miltenyi Biotech) using manufacturers protocol. Single cell suspension was generated from tumors in KPC mice according to the Li *et al* [44]. Non-epithelial progenitor cells were removed using anti-CD31-Biotin (BD Biosciences) and anti-CD45 Biotin (BD Bioscience) using MACS technique. The flowthrough free from the mouse progenitor cells was bound to anti-mouse CD133^−^Microbeads for 10 min on ice and positively purified for CD133^+^ cells by MACS. The purity of separation was tested for each batch by performing a FACS analysis using Anti-CD133^−^PE antibody AC141 (Miltenyi Biotech). The separated populations were used for RNA, Protein and FACS analysis. Cells growing in culture were scraped gently into centrifuge tube and washed once in Wash Buffer (PBS, 0.5% BSA, 2 mM EDTA) before binding to Anti-mouse CD133 microbeads and proceeding as described above.

For labeling with ^13^C glucose, isolated CD133^+^ cells were incubated in glucose free DMEM for 30 min. Following incubation, the cells were transferred to a growth medium (DMEM) with ^13^C glucose (final concentration 25 mM) and incubated further for 30 min. Reaction was quenched and stored in −20°C until further use. CD133^−^ cells were processed in parallel with ^12^C glucose (final concentration 25 mM) added to DMEM.

### Metabolite extraction method

After quenching, samples of each cell type were weighed into and mixed in a single glass vial. To each mixture of ^13^C-labeled (CD133^+^) and ^12^C-labeled (CD133^−^) cell pellets, 400 μL of boiling 75:25 ethanol:water was added. This solution was vortexed and incubated at 95°C for 5 min, then vortexed and incubated at 95°C for an additional 5 min. The solutions were then immediately cooled on ice for 3 min, vortexed, and transferred to a 1.5 mL microcentrifuge tube. To these solutions, 400 mL of LC/MS-grade water was added. The solutions were sonicated for 15 min, vortexed and centrifuged at 12000 rpm for 4 min at 4°C. 400 μL of each supernatant was then filtered through a 0.2 μm Millipore Ultrafree hydrophilic PTFE filtration unit prior to analysis by UPLC/MS^e^.

### UPLC-MS/MS determination of glycolytic intermediates in CD133 extracts

A Waters Acquity UPLC was used to desalt and concentrate glycolytic intermediates prior to introduction to a Waters TQD for MS/MS analysis. Separation was achieved with a Waters Acquity HSS T3 C_18_ column (2.1 mm × 100 mm) heated to 35°C employing a flow rate of 0.35 mL/min and mobile phase A and B, 0.1% aqueous formic acid and 0.1% formic acid in acetonitrile, respectively, and with the following gradient: 0% B, 0 to 2.0 min, 0% B to 10% B, 2.0 to 2.5 min, 10% B to 50% B, 2.5 to 3.5 min, 50% B to 97% B, 3.5 to 4.5 min, 97% B, 4.5 to 5.5 min, 97% B to 0% B, 5.5 to 6.0 min. Total run time is 9 minutes. Electrospray ionization tandem mass spectrometric methods (ESI-MS-MS) were created for eight compounds using MRM transitions optimized by direct infusion. Parameters of the ESI-MS-MS system were selected based on in-source generation of the deprotonated molecular ions of each glycolytic intermediate as well as production of compound-specific fragment ions. The following negative ionization mode parameters were used for determination of glycolytic intermediates in cell extracts: capillary, 3.20 kV; cone, 25 V; extractor, 3 V; rf lens, 0.3 V; source temperature, 150°C; desolvation temperature, 500°C; desolvation gas flow, 800 L/h; cone gas flow, 20 L/h; low-mass resolution (Q1), 15 V; high-mass resolution (Q1), 15 V; ion energy (Q1), 0.3 V; entrance, −1 V; exit, 5 V; low-mass resolution (Q2), 15 V; high-mass resolution (Q2), 15 V; ion energy (Q2), 3.5 V.

Peak area ratios of ^13^C-labeled:^12^C-labeled analytes of interest measured in mixed extracts were used in conjunction with cell count normalization data to determine the ratio of analytes in CD133^+^ versus CD133^−^ cells.

### Glucose uptake assay

Cell based glucose uptake assay kit (Cayman Chemicals) for used for measuring glucose uptake in CD133^+^ and CD133^−^ cells from tumor. For labeling, isolated cells were starved in glucose free media for 30 min prior to labeling with 150 ug/ml 2-NBDG, a fluorescent analog of deoxyglucose. Following incubation for 1 h, the labeled cells were analyzed by flow-cytometry according to manufacturer's instruction.

### Extracellular acidification rate (ECAR) determination

Extracellular acidification rate was determined using the MITO-ID Extracellular pH Sensor probe (Enzo LifeSciences). This probe can be used to detect changes in pH (Extracellular acidification) from cell populations using a 96 well plate system. The probe phosphorescence signal is modulated by pH such that increased acidifications causes increased phosphorescence signal. CD133^+^ and CD133^−^ cells were seeded at a density of 1 × 10^5^/ well and the assay was performed according to manufacturer's instruction.

### Enzymatic assays for glycolysis and TCA cycle

Assays for enzymes in the glycolysis pathway like hexokinase 2 and lactate dehydrogenase and estimation of glycolysis and TCA cycle intermediates like lactate, pyruvate, alpha keto-glutarate and fumarate were done using assay kits from Sigma-Aldrich according to manufacturer's instruction. Experiments were repeated at least 3 times.

### Mitochondrial activity assay

Enzyme assays for mitochondrial complex 1 and 4 were done on isolated mitochondrial lysate using the Mitochondrial Complex 1 and Mitochondrial Complex 4 assay kits (Abcam) according to manufacturer's instruction.

### Immunohistochemistry and immunofluorescence

For immunohistochemistry, paraffin tissue sections were deparaffinized in xylenes and hydrated through graded ethanol. Hematoxylin and Eosin (H&E) staining were used for evaluation of histological features. Slides were steamed with Reveal Decloaker pH9.0 (Biocare Medical) for antigen retrieval of GLUT1 antibody (Sigma) and CD133 antibody. Sniper Universal Blocking Sera (Biocare Medical) were used throughout the protocol. Primary antibodies were diluted according to vendor's instruction and incubated overnight at 4°C. The primary antibody was omitted for the negative controls. For immunofluorescence, fluorescent antibody conjugates were used after primary antibody staining. Slides were counterstained with DAPI and visualized in a Nikon fluorescent microscope. Tissue samples were incubated with mouse IgG1 isotype controls (BD Biosciences) and did not demonstrate any specific staining.

### Pimonidazole labeling of hypoxic regions in pancreatic tumor

Pimonidazole hydrochloride (30 mg/ml in sterile saline) was injected into animals intraperitoneally 6 hours before euthanizing. Fixed tissues were processed as described above. Sections were stained with anti-PDZ antibody according to manufacturers instruction.

### HIF1A activity assay

HIF-1α DNA binding activity was assessed using the HIF-1α Transcription Factor Assay Kit (Cayman Chemical). Briefly, a dsDNA sequence containing the HIF-1α response element (5′-ACGTG-3′) is immobilized to the wells of a 96-well plate. Protein extracts from cells or tumors were equilibrated in the wells and active HIF-1α was free to bind to the HIF-1α response element. After washing, HIF-1α was detected using anti-HIF-1α antibody followed by a secondary antibody conjugated to HRP. The results were analyzed by measuring the absorbance at 450 nm. The samples were normalized by their relative protein concentration.

### *In vivo* activity assay for mitochondrial complex IV

Cryostat sections of pancreatic tumor tissue (7 or 10 μm) were prepared and stored at −80°C until use. For the COX activity staining, frozen sections were brought to room temperature, washed for 5 min with 25 mM sodium phosphate buffer, pH 7.4, and then incubated for 0.5, 1 or 2 h at 37°C with the COX incubation mixture. The COX solution consisted of 10 mg Cytochrome *C* (cat# C7752, Sigma-Aldrich), 10 mg 3,3 diaminobenzidine tetrahydrochloride hydrate (cat# D5637, Sigma-Aldrich) and 2 mg catalase (cat# C1345, Sigma-Aldrich) dissolved in 10 ml of 25 mM sodium phosphate buffer. The solution was filtered after preparation and the pH was adjusted to 7.2–7.4 with 1 N NaOH.

For double labeling, sections were brought to room temperature, washed for 10 min with 25 mM sodium phosphate buffer and then incubated for 1.5 h at 37°C with the COX incubation mixture. The reaction was stopped by washing in 25 mM sodium phosphate buffer for 5 min. Sections were then fixed with 10% formalin for 10 min at room temperature. Sections were then washed with PBS and blocked for endogenous biotin using a biotin blocking kit (Dako X0590), followed by blocking with 10% goat serum in PBS for 1 h. Rabbit anti-CD133 antibodies were applied at a dilution of 1:200 overnight at 4°C. Antibody binding was detected using a biotinylated goat anti- rabbit IgG (Vector Labs) followed by streptavidin-alkaline phosphatase (Jackson ImmunoResearch). Immunoreactivity was then detected using the Vector Red Alkaline Phosphatase Substrate Kit I (Vector #SK-5100) and sections were counterstained with hematoxylin, dehydrated, cleared with xylene and mounted with permount.

### Data representation and statistical analysis

Isolation of CD133^+^ and CD133^−^ cells were done from at least 4–5 tumors. All experiments were performed at least 3–4 times from independent sorts of CD133^+^ and CD133^−^ cells. Values are expressed as the mean ± standard error of the mean (SEM). The significance of the difference between the control and each experimental arms was analyzed by the unpaired Student's *t* test and *P* < 0.05 was considered statistically significant.

## SUPPLEMENTARY MATERIALS FIGURES


