# Supplementary material for: Microenvironment mediated alterations to metabolic pathways confer increased chemo-resistance in CD133+ tumor initiating cells
Source: Oncotarget. 2016 Jul 26;7(35):56324–37. doi: 10.18632/oncotarget.10838 (PMC5302917; doi:10.18632/oncotarget.10838)
Supplement: Supplementary file 1 [file oncotarget-07-56324-s001.pdf]

Microenvironment mediated alterations to metabolic pathways confer increased chemo-resistance in CD133^{+} tumor initiating cells

Supplementary Materials

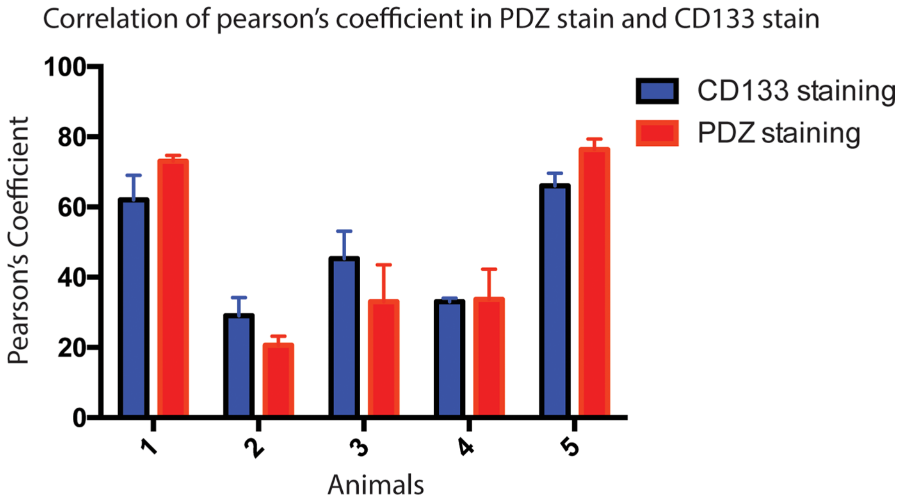

Supplementary Figure S1: Bar graph representing Pearson's Coefficient of PDZ staining and CD133 in pancreatic tumors.

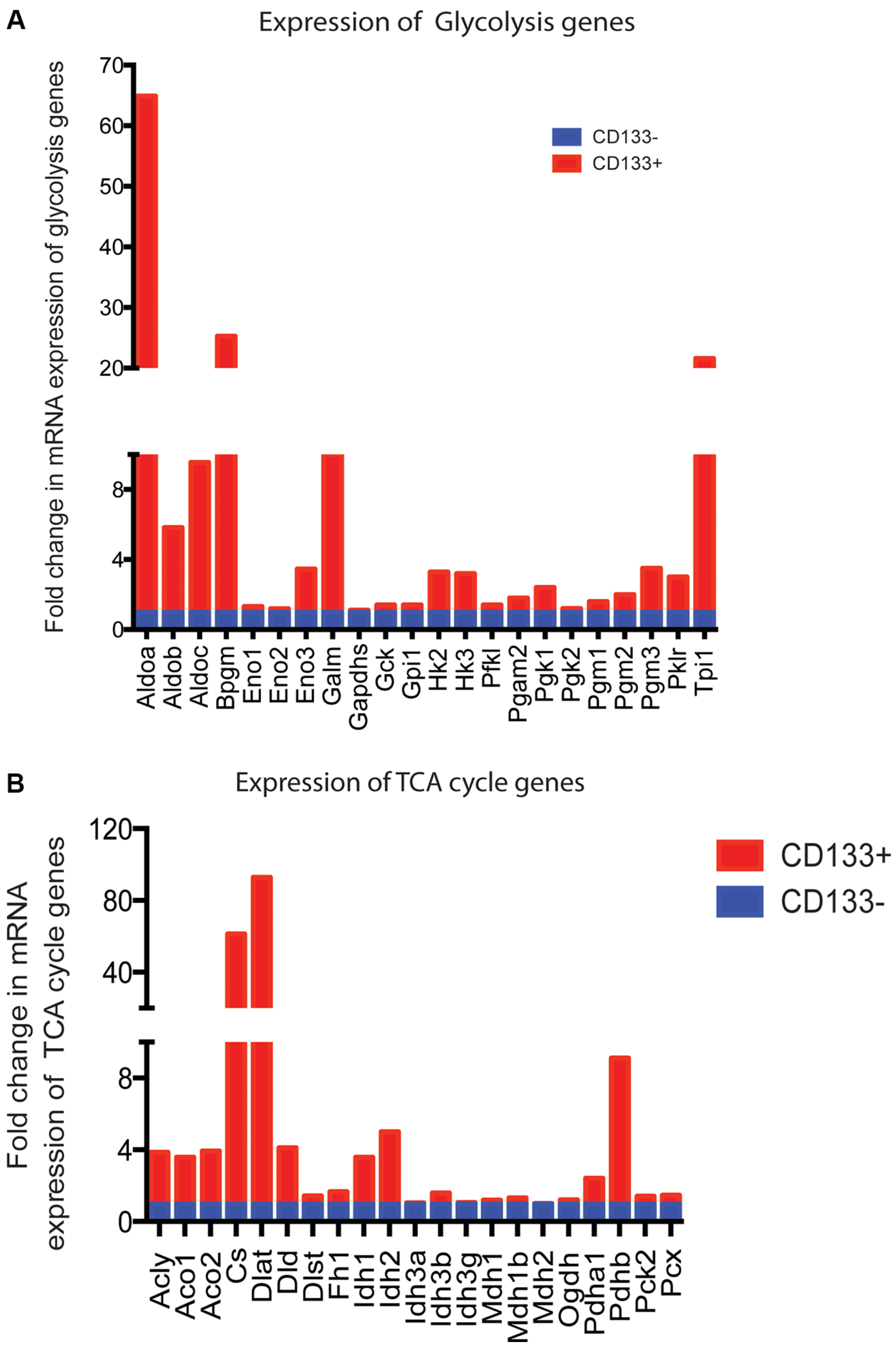

Supplementary Figure S2: CD133^{+} cell have higher metabolic gene expression. CD133^{+} cells had increased expression of several glycolysis related genes (**A**) and TCA cycle genes (**B**), as compared to CD133^{-} cells.

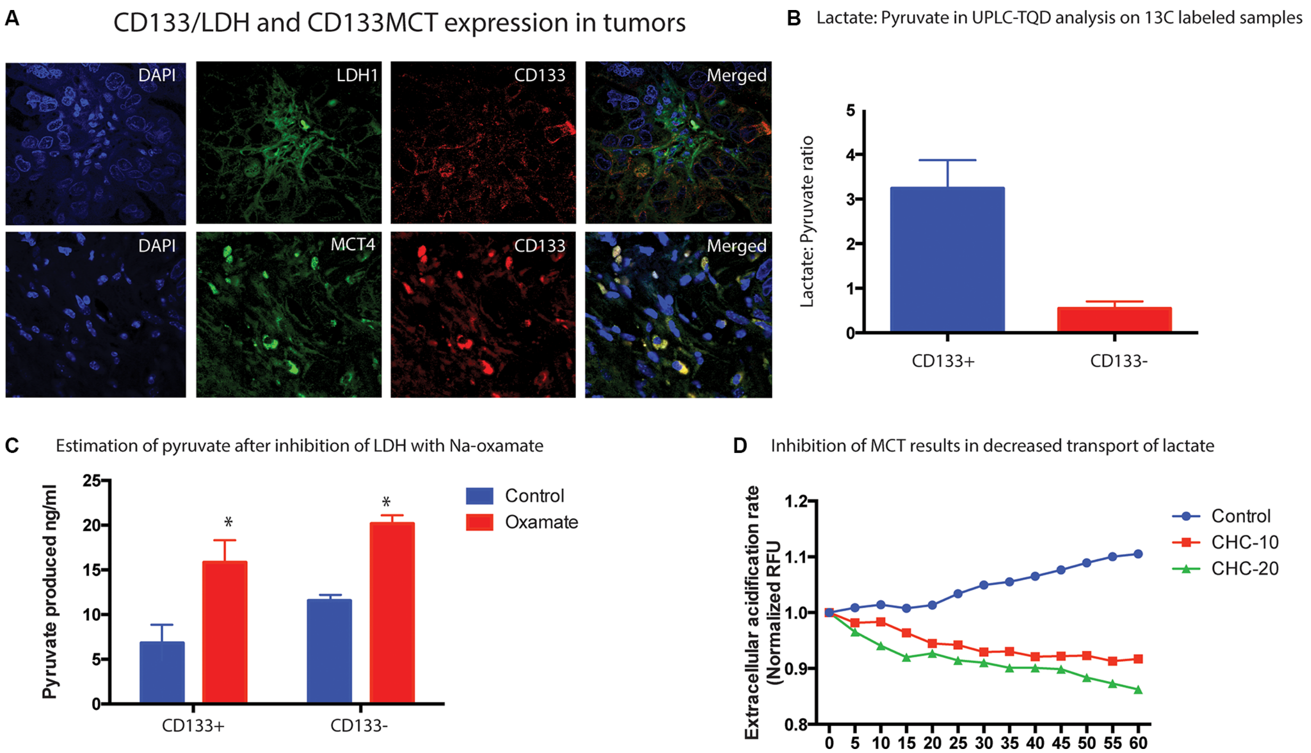

Supplementary Figure S3: CD133 cells have increased expression of lactate synthesis and transport genes. CD133^{+} cells co-stain for LDHA and MCT4 (**A**). Increased lactate pyruvate ratio is observed in the UPLC-TQD labelling studies (**B**) . Inhibition of MCT by an inhibitor, CHC results in lower extracellular acidification rate (ECAR) indicating accumulation of lactate in cells (**C**).

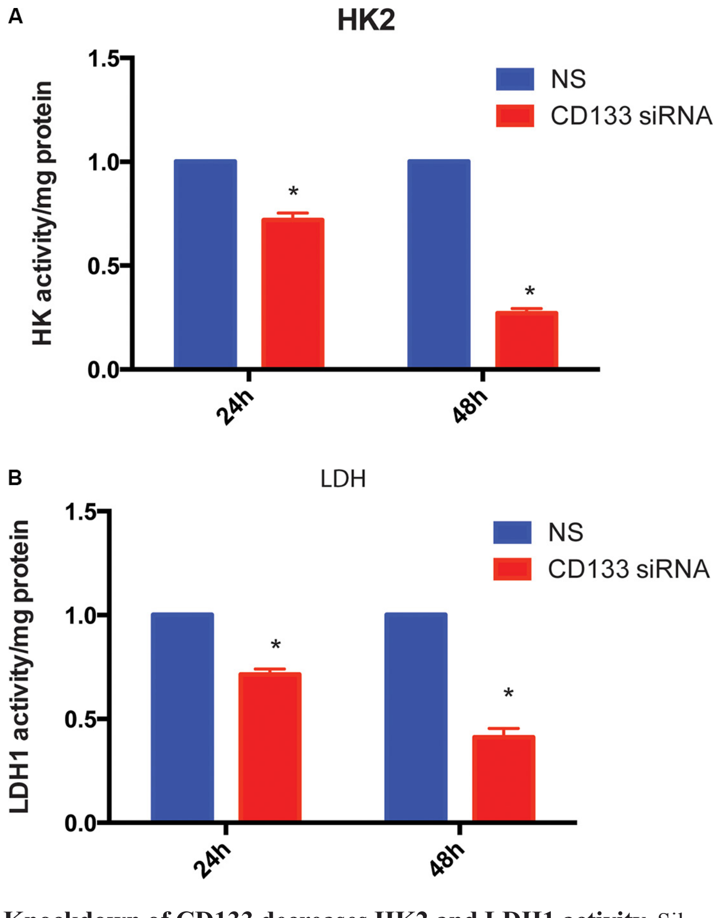

Supplementary Figure S4: Knockdown of CD133 decreases HK2 and LDH1 activity. Silencing CD133 decreases activity of HK2 (**A**) and LDH1 (**B**) in a time dependent manner.

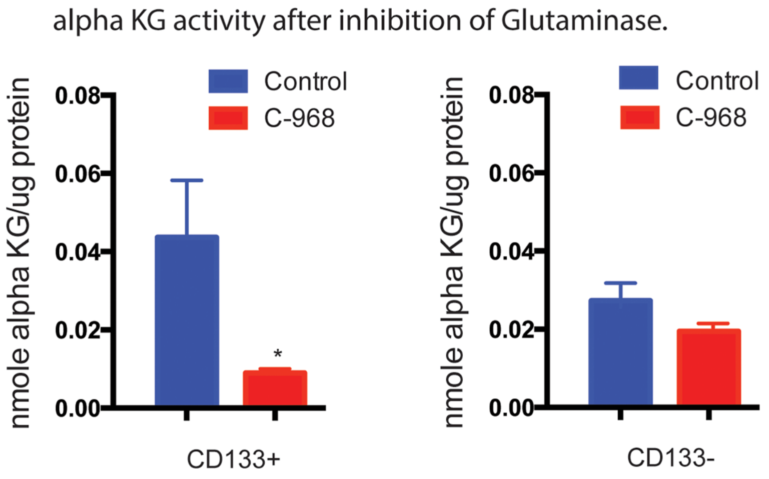

Supplementary Figure S5: Glutaminase inhibition decreases alpha-ketoglutarate accumulation in CD133^{+} cells. Treatment by C-968 decreases levels of alpha-ketoglutarate in CD133^{+} cells, with no significant change in CD133^{-} cells.

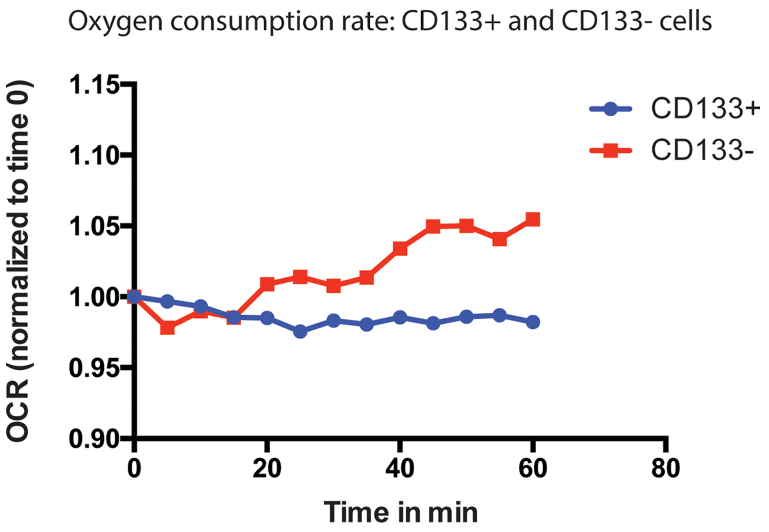

Supplementary Figure S6: Oxygen Consumption Rate of CD133 positive and CD133^{-} cells using the MITO-ID Extracellular O2 Sensor kit. This kit contains a phosphorescent oxygen sensitive reagent enabling high-throughput and real-time oxygen consumption readout. In this assay, MITO-ID® Extracellular O_2 SensorProbe is quenched by oxygen, through molecular collision, and thus the amount of fluorescence signal is inversely proportional to the amount of extracellular oxygen in the sample. Rates of oxygen consumption are calculated from the changes in fluorescence signal over time. The CD133^{+} cells have low OCR compared to CD133^{-} cells.
